# Pharmacist workforce training in pharmacogenomics with a focus on rural and underserved areas

**DOI:** 10.3389/fgene.2026.1794122

**Published:** 2026-03-30

**Authors:** Moataz Mohamed, Pamala A. Jacobson, Alyssa Johnson, Jacob Tyler Brown

**Affiliations:** 1 Experimental and Clinical Pharmacology, College of Pharmacy, University of Minnesota, Minneapolis, MN, United States; 2 Pharmacy Practice and Pharmaceutical Sciences, College of Pharmacy, University of Minnesota, Minneapolis, MN, United States

**Keywords:** education, pharmacogenetics, pharmacogenomics, rural, underserved

## Abstract

**Objective:**

Our primary objective is to demonstrate the benefits of pharmacogenomic (PGx) education for pharmacists that serve rural and underserved communities through pre- and post-surveys assessing their overall change in knowledge and attitudes towards PGx testing.

**Methods:**

Participants completed a 16-week, asynchronous didactic course, attended in-person case-based PGx presentations from content experts, and viewed materials from a two-day international PGx conference. Pre- and post-surveys designed to measure changes in self-assessed knowledge, confidence, and attitudes towards PGx testing were completed by all participants. All participants who had completed the program were also invited to take a longitudinal survey to assess long-term program impacts.

**Results:**

Twenty-three participants located throughout the state of Minnesota completed the program. Participant’s confidence in their PGx knowledge increased significantly from the beginning of the program to the end. In the longitudinal survey, nearly all participants (93%) responded “yes” when asked if the program influenced their perspectives on the importance of PGx in pharmacy and healthcare, and nearly 75% responded “yes” when asked if the PGx certificate program had contributed to their professional growth or career advancement. The majority (87%) reported that they had shared the knowledge gained from the program with other healthcare providers.

**Conclusion:**

These findings show a high level of impact on PGx knowledge in pharmacists practicing in rural and underserved areas, and highlight increased likelihood of PGx implementation after the education program.

## 1 Introduction

Although pharmacogenomic (PGx) testing has seen a considerable increase in use over the past several years (Luczak et al., 2021a), most healthcare professionals lack sufficient understanding of PGx to confidently implement testing in their practice, order testing and make recommendations based on results (Wondrasek et al., 2024). PGx educational programs, PGx post-graduate year 2 pharmacy residencies, and increased PGx education within pharmacy school curricula are gradually addressing this knowledge gap; however, few, if any, PGx educational programs offered to working healthcare professionals have focused on those working with rural and/or underserved populations. Underserved communities are at risk of worsening health disparities due to less availability of PGx testing, a lack of understanding on how to interpret genetic variants present (particularly those less well studied and present in specific ancestry populations), challenges in identifying when to use testing in their population and/or clinic setting, and fewer healthcare resources that limit exploration of new and innovative tools for medication management (Luczak et al., 2021b; Magavern et al., 2022). Focusing PGx educational programs towards rural and underserved populations will help in two ways. First, it will provide the necessary knowledge and tools to those who are in position to recommend, order, and interpret PGx testing for individuals where access to PGx experts or clinics is limited or nonexistent. Second, it will extend the reach of PGx testing beyond the large academic and private health systems where it is predominantly being implemented (Luczak et al., 2021a).

A recent systematic review comprehensively examined responses from over 12,000 pharmacists’ and pharmacy students’ knowledge and attitudes towards PGx from twenty-six countries, predominantly from the United States (Wondrasek et al., 2024). Broadly speaking, this review found the majority of pharmacists and pharmacy students to have positive views towards PGx while also desiring education in multiple formats (e.g., lectures, workshops, online continuing education), reinforcing the need for tailored PGx training programs delivered in ways that encourage and allow practicing pharmacists in resource limited communities to participate.

Our previous article summarized the results of participants’ PGx knowledge and attitudes after completion of a PGx workforce training program through the University of Minnesota College of Pharmacy for pharmacists practicing in rural and underserved areas (Brown et al., 2022). Building on this prior work, the primary objective of this article is to demonstrate the benefits of PGx education for actively working pharmacists that serve rural and underserved communities through pre- and post-surveys assessing their overall change in knowledge and attitudes towards PGx testing. The secondary objective is to describe the longitudinal impact in PGx utilization and attitudes for all participants who had completed the program.

## 2 Methods

### 2.1 Study design

The University of Minnesota College of Pharmacy offered a competitive application based PGx training program funded by the Minnesota Department of Health to pharmacists licensed in Minnesota actively working in and with rural and/or underserved populations. After selection and over the course of 1 year, participants completed a 16-week, asynchronous didactic course, a full-day of interactive in-person case-based PGx presentations from content experts, viewed materials from a two-day international PGx conference hosted by the University of Minnesota, and attended at least one PGx Extension of Community Health Outcomes (ECHO) session (Jacobson et al., 2026) hosted by the College of Pharmacy. The full details of recruitment, the program, topics covered, and learning objectives are described in a previous publication (Brown et al., 2022). This manuscript builds upon this initial analysis by including both pre- and post-survey assessments as well as a longitudinal survey of participants from each of the two cohorts that have completed the program. Learners were reimbursed a small stipend for their participation in the program and for travel expenses incurred for the in-person workshop focused on PGx clinical cases. The study (STUDY00014431) was classified as “Not Human Research” by the University of Minnesota Institutional Review Board and IRB approval was not required.

### 2.2 Surveys

This manuscript evaluates outcome in our second cohort of participants to complete the PGx certificate training where participants completed pre- and post-surveys through Qualtrics (Qualtrics, Provo, UT) designed to measure changes in their self-assessed knowledge, confidence, and attitudes towards PGx testing. Our first cohort only completed a post-training survey and these survey results were compared between the first (n = 12, 2022) and second (n = 23, 2023) cohorts to compare the two experiences (Supplementary Material).

An additional longitudinal post-program completion survey through Qualtrics was conducted approximately 2 years after the first cohort had completed their training and 1 year after the second cohort had completed training to assess longer term impacts of this certificate program on participants and their health system’s utilization of PGx testing (Supplementary Material).

### 2.3 Statistical analysis

Participants’ demographics and survey responses were summarized using frequencies and percentages. The analyzed survey questions were in Likert scale and therefore Wilcoxon signed rank test was used to compare the pre- and post-survey responses of the second cohort (2023), while Wilcoxon rank sum test was used to compare post-survey responses of the first (2022) and second (2023) cohorts. A p-value <0.05 was considered statistically significant. All analyses and plots were conducted using R (version 4.3.0, Vienna, Austria) on RStudio software (version 2023.6.1.524, Massachusetts, US).

## 3 Results

### 3.1 Second cohort demographic/practice settings

Twenty-three participants located throughout the state of Minnesota (Supplementary Figure S1) completed the program, 21 of which held Doctor of Pharmacy degrees and 2 with Bachelor of Science in Pharmacy degrees, Table 1. Slightly more than half (n = 12, 52.2%) earned their pharmacy degree after 2010, around a quarter (n = 6, 26.1%) between 2006–2010, and five (21.7%) before 2006. The majority of participants worked in either a hospital (n = 9) or retail (n = 9) setting, while seven worked in a clinic. One participant worked in a home infusion setting, one as a medication therapy management manager, and one described their setting as a long-term care independent rural pharmacy.

**TABLE 1:** Demographic and pharmacogenomic clinical practice experience of the second cohort.

Demographics/Questions		Responses n (%) [n total = 23]
Degree		
​	BSPharm	2 (8.7)
​	PharmD	21 (91.3)
Year earned the pharmacy degree		
​	Before 2006	5 (21.7)
​	2006–2010	6 (26.1)
​	After 2010	12 (52.2)
Current clinical practice setting (can pick more than one)		
​	Hospital	9 (39)
​	Retail pharmacy	9 (39)
​	Clinic	7 (30)
​	Other	4 (17)
Has your clinical practice implemented a PGx program?		
​	Yes, a pharmacy-led program	3 (13)
​	We are currently in the process of implementing a PGx program	6 (26.1)
​	Yes, a medicine- or genetic counselor-led program	0 (0)
​	No	14 (60.9)
Use of PGx in the participant’s organization		
​	Yes	4 (17.4)
​	No	16 (69.6)
​	Don’t know	3 (13.0)
Number of PGx tests done annually in the participant’s organization		
​	100 or more	1 (4.3)
​	11 to 50	1 (4.3)
​	Fewer than 10	12 (52.2)
​	Don’t know	9 (39.1)
How often do you personally use PGx-guided care in your practice?		
​	Daily	1 (4.3)
​	Infrequently	1 (4.3)
​	Never	21 (91.3)
Do you expect your organization will increase its use of PGx testing services over the next 3 years?		
​	Definitely yes	4 (17.4)
​	Might or might not	5 (21.7)
​	Probably yes	14 (60.9)
Did you find the PGx extension of community health outcomes session to be beneficial to your learning?		
​	Definitely yes	13 (56.5)
​	Might or might not	4 (17.4)
​	Probably not	1 (4.3)
​	Probably yes	5 (21.7)

Three participants (13%) were part of a health system that had partially implemented a PGx program or implemented it in clinics that the trainee was not practicing, six (26%) were currently in the process of implementing a PGx program but were not part of the implementation, while the majority of participants’ organizations had not yet implemented PGx (n = 14; 61%). When asked if their organization used PGx in clinical care, four respondents said “yes” (17.4%), 16 said “no” (69.6%), and three (13%) were unsure. Over 50% (n = 12) of participants’ organizations ordered fewer than 10 PGx tests per year, while around 40% (n = 9) said they did not know how many PGx tests their organization ordered per year, Table 1.

### 3.2 Second cohort pre vs. post survey results

**FIGURE 1:**
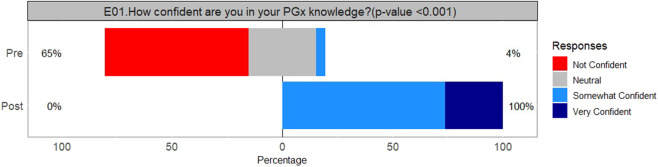
Assessing second cohort (2023) participant’s confidence in their PGx knowledge before and after completing the PGx program (n = 23).

**FIGURE 2:**
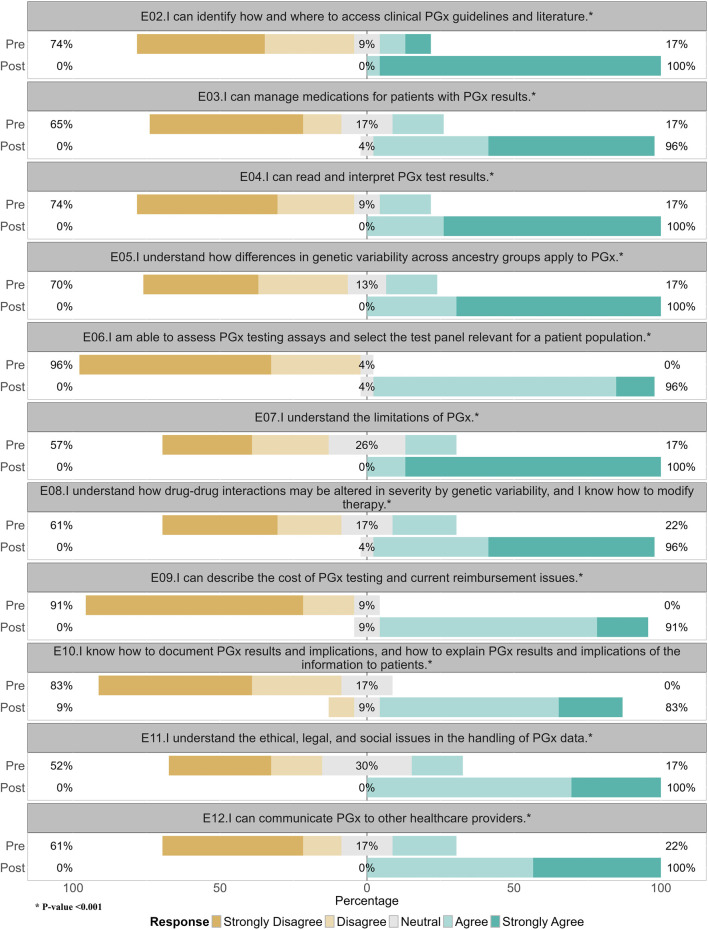
Pre and post completion of program assessment of participant’s pharmacogenomic knowledge (second cohort 2023, n = 23).

Participant’s confidence in their PGx knowledge increased significantly (Wilcoxon signed rank test, p < 0.001) from the beginning of the program to the end, Figure 1. Figure 2 illustrates participants’ changes in PGx knowledge, attitudes, and confidence in interpreting and applying PGx results prior to starting and immediately after completion. All participants indicated they had greater confidence in accessing PGx guidelines and literature, understanding the limitations of PGx, and communicating PGx to other healthcare providers by the end of the program. The only question that did not have a greater than 90% agree or strongly agree response was in regard to documenting PGx results and implications/how to explain PGx results to patients, although this was still 83% in agreement.

When participants were asked about what barriers in their clinical practice may hinder their ability to implement PGx, cost, reimbursement, and a lack of time were noted by several individuals. Additionally, the following are select comments from participants:

“Great information, but more importantly, great skill-building course to be able to apply PGx information to clinical practice.”

“I enjoyed this course immensely! It was informative and fun to connect with pharmacists from across the state. Overall, I felt it was a great use of my time and look forward to following the development and implementation of PGx programs at my site and beyond.”

“I am grateful for this opportunity to learn about PGx from experts in the field. PGx is such a complex area and I thought you did a really excellent job of highlighting all the various topics of consideration that one needs to understand. I have enjoyed taking the course and have promoted it wildly among my pharmacist friends and co-workers.”

“I do not feel like an expert in the field, but I feel way more comfortable with the field than I did before I took the course. I will use it to continue to grow my knowledge.”

### 3.3 Post-program completion survey comparison between first and second cohorts

**FIGURE 3:**
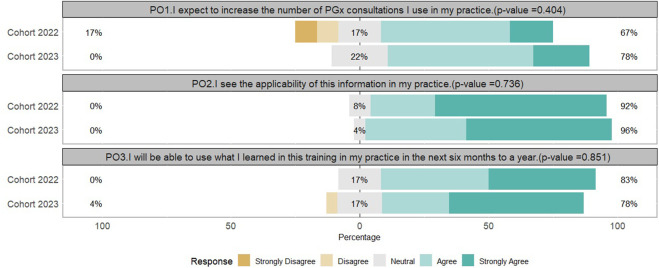
Program outcome questions and responses of the first “2022” (n = 12, 2022) and second “2023” (n = 23, 2023) cohorts from the post survey.

Participants from each cohort answered three questions on the program’s impact at the end of the program on their future implementation or utilization of PGx, Figure 3. Most of the participants in the first and second cohorts reported that they expect to increase the number of PGx consultations after completing the program, they saw how this information applied to their practice, and that they would be able to use what they learned in the next 6 months to 1 year. None of the participants, in either cohort, had negative attitudes towards the applicability of the information learned in the program in their practice; however, 17% of both cohorts indicated that they were neutral and 4% (n = 1, second cohort) disagreed that they would be able to use what they learned in the next 6 months to 1 year. The responses of both cohorts to these three questions did not differ statistically, Figure 3. Responses were not significantly different when compared to our previously published data on the first and second cohort post-program survey questions pertaining to their overall PGx knowledge, Supplementary Figure S2.

### 3.4 Longitudinal post-program completion survey in first and second cohorts

Fifteen participants completed all aspects of the longitudinal survey while one participant completed part of it, which was 1 year post completion of the program for the second cohort (2023) and 2 years post completion for the first cohort (2022). Four (25%) were from the first cohort and 12 (75%) from the second. Thirteen (81%) held a Doctor of Pharmacy degree, one (6%) held Bachelor of Science in Pharmacy and Doctor of Pharmacy degrees, and two (13%) had a Bachelor of Science in Pharmacy degree. The year pharmacy degree was earned was 25% (n = 4) before 2006, 19% (n = 3) from 2006 to 2010, and 56% (n = 9) after 2010.

Seven (44%) participants stated their organization uses PGx in clinical care, although only 19% have a pharmacy-led program or are in the process of implementing a PGx program. Nearly two-thirds (63%) responded “probably yes” or “definitely yes” when asked if they expected their organization to increase their use of PGx testing over the next 3 years, while two-thirds reported that their understanding of PGx has continued to improve since completing the program, with all participants either somewhat or strongly agreeing that they feel confident interpreting PGx test results and applying them to clinical decision-making. The majority (87%) somewhat or strongly agreed when asked if they are more likely to recommend PGx testing for their patients as a result of the PGx certificate program. Nearly all participants (93%) responded “yes” when asked if the program influenced their perspectives on the importance of PGx in pharmacy and healthcare, and nearly 75% responded “yes” when asked if the PGx certificate program had contributed to their professional growth or career advancement. Lastly, 87% reported that they had shared the knowledge gained from the program with other healthcare providers.

## 4 Discussion

PGx education of the healthcare workforce is crucial to achieving broad implementation and utilization of PGx results to guide medication dose and selection, with the aim of avoiding adverse effects and reducing the time to response. Although current pharmacy students and more recent graduates receive some PGx education in their curricula, it varies between schools and the majority of practicing pharmacists have not had adequate education or experience with PGx. The goal of this PGx workforce training program was to specifically target healthcare providers working with rural and underserved populations, who often face geographical isolation and limited access to educational resources. By providing targeted education program aimed to improve accessibility and implementation of PGx across all patient populations.

This program was tailored by the University of Minnesota College of Pharmacy to fit the needs of the healthcare workforce in Minnesota in regards to where people live and who those people are. The state’s population of nearly 6 million people is spread out across a predominant metropolitan area, suburbs, and other non-urban/rural areas (Bishop et al., 2021). Minnesota’s non-white population also requires healthcare providers to thoughtfully order from PGx testing companies with inclusive panels to account for more potential variants. Unfortunately, Minnesota also has some of the worst healthcare disparities between white and non-white populations. It is our goal that by expanding the reach of pharmacists with PGx training in Minnesota we will in turn increase the likelihood of PGx testing being available to both non-white individuals and/or those living outside of large, metropolitan areas.

Participants in the second cohort of this PGx training program showed dramatic improvement in their knowledge, confidence, and understanding of PGx after completion as compared to before. Additionally, participants who had completed the program from the second cohort had similar positive post-completion survey responses as compared to the first cohort (Brown et al., 2022). These pharmacists represented a variety of practice settings, including community, hospital, and long-term care, in rural and/or underserved areas, bringing PGx proficiency to patient populations that historically have not been a primary focus of PGx testing. While most pharmacists responded that their institution had not yet implemented PGx testing, over half expected this to increase over the next 3 years.

Complete reviews of PGx educational programs are described elsewhere (Kisor and Farrell, 2019). Of note, an educational program consisting of 11 PGx lectures in Canada that was offered both synchronously and asynchronously showed improved confidence and competency by the pharmacists who completed it (Hayashi et al., 2022). Additionally, a three part PGx webinar series offered at a healthcare system in the United States showed similar improvements (Lee et al., 2022). This program is novel in that, 1) it focuses on pharmacists practicing in rural and underserved areas, 2) the funding mechanism allowed for participants to receive a stipend for completing the program, and 3) participants had to apply and be selected. A noted weakness in our report of the first cohort was the lack of a formal pre-program evaluation and direct comparison of their own PGx knowledge pre- and post-training completion, which limited our ability to fully assess the effect of the program. However, the second cohort had formal pre- and post-program evaluations. We were able to show significant overall improvements related to understanding and knowledge of PGx in participants from the start of the program to completion in the second cohort, and showed similar post-completion survey responses in both cohorts.

PGx continues to see increased implementation at both academic and non-academic health systems, and is currently under consideration from the Board of Pharmacy Specialties to be added to their list of post-licensure certifications (BPS, 2025). Although not all pharmacists utilizing PGx in their practice would necessarily pursue a board certification in PGx, educational programs such as the one described herein would be a logical step for a pharmacist looking to increase their competency in PGx and/or attain PGx board certification.

We surveyed all individuals who had completed the program either one or 2 years later with findings suggesting that they retained PGx interest/knowledge, shared their own PGx knowledge with other healthcare professionals, and that it empowered them to recommend PGx testing for their patients. These responses are suggestive that pharmacists undergoing educational programs in PGx continue to utilize this knowledge and discuss it as a medication guiding option with other healthcare professionals. Some participants also continue to attend PGx ECHO sessions offered through the University of Minnesota College of Pharmacy (Jacobson et al., 2026), showing continued interest in PGx among these learners. However, the real long-term impact of PGx educational programs is difficult to measure, as individuals regularly change positions, leave the workforce, or may not be in a position with their employer to implement PGx testing.

The study comes with limitations. First, the questionnaires were self-reported and hence they could be subjected to social desirability bias. Second, enrollment of participants from rural areas is challenging resulting in a small sample size of our cohort which could limit the generalizability of the results, which was also true of our longitudinal survey results which were more heavily weighted towards individuals who had more recently completed the program.

## 5 Conclusion

These findings suggest a high level of impact on PGx knowledge in pharmacists practicing in rural and underserved areas, and highlight increased likelihood of PGx implementation after PGx education program, advocating for additional training opportunities.

## Data Availability

The raw data supporting the conclusions of this article will be made available by the authors, without undue reservation.

## Supplementary material

SUPPLEMENTARY FIGURE 1 Participants located throughout Minnesota mapped by zip code.

SUPPLEMENTARY FIGURE 2 Comparing overall PGx knowledge between first and second cohorts in the post-program survey questions.
