# Supplementary material for: Pharmacist workforce training in pharmacogenomics with a focus on rural and underserved areas
Source: Front Genet. 2026 Mar 30;17:1794122. doi: 10.3389/fgene.2026.1794122 (PMC13070540; doi:10.3389/fgene.2026.1794122)
Supplement: Supplementary file 1 [file DataSheet2.pdf]

UNIVERSITY OF MINNESOTA
Driven to Discover®

Default Question Block

Pharmacogenomics (PGx) Clinical Workforce Training Program Follow-Up Survey

We are seeking long term follow-up and feedback on your experience with the PGx clinical workforce training program and whether it has resulted in practice changes. We have developed a brief survey aiming to help us understand if these types of educational programs are valuable. This will help us to determine if we should pursue offering additional training opportunities.

The survey should take no more than five minutes to complete. We greatly appreciate your feedback.

What is your name?

What is the email you used to register for the course?

When did you begin the PGx training program?

- ○ January 2022
- ○ January 2023

Please select your degree(s): (please select all that apply)

- ☐ BSPharm
- ☐ PharmD
- ☐ MS

What year did you earn your pharmacy degree?

Block 1

Has your clinical practice setting changed since participating in the program?

- ○ Yes
- ○ No

What is your current clinical practice setting? (Please select all that apply)

- ☐ Hospital
- ☐ Retail pharmacy
- ☐ Clinic
- ☐ Managed care
- ☐ [ ] Other

What is the zip code of your clinical practice?

[ ]

Has your clinical practice implemented a PGx program?

- No
- Yes, a pharmacy-led program
- Yes, a medicine or genetic counselor-led program
- We are currently in the process of implementing a PGx program

Does your organization use PGx in clinical care?

- Yes
- No
- I don't know

Approximately how many PGx tests does your organization order per year?

- Fewer than 10
- 11 to 50
- 51 to 99
- 100 or more

How often do you personally use PGx-guided care in your practice?

- Daily
- Weekly
- Monthly

- Infrequently
- Never

Do you expect your organization will increase its use of PGx testing services over the next three years?

- Definitely not
- Probably not
- Might or might not
- Probably yes
- Definitely yes

Block 2

Has your understanding of PGx continued to improve since completing the program?

- Yes (please describe how it has improved and / or what did you do to improve knowledge)

- ○ No (please describe what barriers you have encountered in improving your PGx knowledge)

Please rate the following statement: I feel confident interpreting PGx test results and applying them in clinical decision-making.

- ○ Strongly agree
- ○ Somewhat agree
- ○ Neither agree nor disagree
- ○ Somewhat disagree
- ○ Strongly disagree

Please rate the following statement: I am confident in discussing PGx with patients and colleagues.

- ○ Strongly agree (please explain)

○ Somewhat agree (please explain)

○ Neither agree nor disagree (please explain)

○ Somewhat disagree (please explain)

○ Strongly disagree (please explain)

Have you developed any new strategies for explaining PGx test results and their implications to patients?

○ Yes (please describe new strategies)

○ No

Have you been able to incorporate PGx information into your clinical practice more frequently as a result of this program?

- Yes
- No (please describe the reasons / barriers to why you have not incorporated PGx testing more)

What additional training opportunities / support would you need to further PGx implementation in your practice?

Can you provide examples of how you have used PGx knowledge in patient care?

What challenges, if any, have you faced in applying PGx in your practice?

Have you observed any changes in patient outcomes as a result of using PGx information in your practice?

- Yes (please describe what changes in patient outcomes have you observed)
- No

Please rate the following statement: PGx testing has been favorably perceived by providers that I work with.

- Strongly agree
- Somewhat agree
- Neither agree nor disagree
- Somewhat disagree
- Strongly disagree

Please rate the following statement: PGx testing has been favorably perceived by my patients.

- Strongly agree
- Somewhat agree
- Neither agree nor disagree
- Somewhat disagree
- Strongly disagree

Please rate the following statement: As a result of the PGx certificate program I am more likely to recommend PGx testing for my patients.

- Strongly agree
- Somewhat agree
- Neither agree nor disagree
- Somewhat disagree
- Strongly disagree

Have you shared the knowledge gained from the program with any physicians, pharmacists, or other providers?

- Yes (please describe)
- No

Has the PGx certificate program contributed to your professional growth or career advancement?

- Yes (please describe how it has contributed to your professional growth or career advancement)
- No

Have you pursued additional education or training in PGx or related fields as a result of this program?

- Yes
- No

Did the program influence your perspectives on the importance of PGx in pharmacy and healthcare?

- Yes (please describe how the program influenced your perspective)
- No

Powered by Qualtrics
