# Supplementary material for: Pharmacist workforce training in pharmacogenomics with a focus on rural and underserved areas
Source: Front Genet. 2026 Mar 30;17:1794122. doi: 10.3389/fgene.2026.1794122 (PMC13070540; doi:10.3389/fgene.2026.1794122)
Supplement: Supplementary file 2 [file DataSheet1.pdf]

Demographic Data

Thank you for taking this post-assessment for the course "Applied PGx Education for the Healthcare Professional." This information will help us assess the course so we are effectively helping pharmacists learn about pharmacogenomics and how to incorporate it into their practices.

The survey will take about 5-7 minutes.

First, we'd like to ask a few questions about you.

What is the email address that you used to register for the course?

____

Select which degree(s) you have.

- ○ BSPharm
- ○ PharmD
- ○ MS

What year did you earn your pharmacy degree?

____

Now we'd like to ask you a little about your current clinical practice.

What is your current clinical practice setting? (You may pick more than one)

- ☐ Hospital
- ☐ Retail Pharmacy
- ☐ Clinic
- ☐ Managed Care
- ☐ ______ Other

What is the zip code of your clinical practice?

______

Has your clinical practice implemented a PGx program?

- ☐ No
- ☐ Yes, a pharmacy-led program
- ☐ Yes, a medicine- or genetic counselor-led program
- ☐ We are currently in the process of implementing a PGx program

Does your organization use PGx in clinical care?

- ○ Yes
- ○ No
- ○ I don't know

Approximately how many PGx tests does your organization order per year?

- Fewer than 10
- 11 to 50
- 51 to 99
- 100 or more
- I don't know

How often do you personally use PGx-guided care in your practice?

- Daily
- Weekly
- Monthly
- Infrequently
- Never

Do you expect your organization will increase its use of PGx testing services over the next three years?

- Definitely yes
- Probably yes
- Might or might not
- Probably not
- Definitely not
- I don't know

Course Objectives

Now we'd like to ask you about your experience with the course "Applied PGx Education for the Healthcare Professional."

How confident are you in your PGx knowledge?

- ○ Very confident
- ○ Somewhat confident
- ○ Neither confident nor not confident
- ○ Not confident

Answer the following set of questions to indicate how you feel today, after completing this course.

	Strongly agree	Somewhat agree	Neither agree nor disagree	Somewhat disagree	Strongly disagree
I can identify how and where to access clinical PGx guidelines and literature.	○	○	○	○	○
I can manage medications for patients with PGx results.	○	○	○	○	○
I can read and interpret PGx test results.	○	○	○	○	○
I understand how differences in genetic variability across ancestry groups applies to PGx.	○	○	○	○	○
I am able to assess PGx testing assays and select the test panel relevant for a patient population.	○	○	○	○	○

	Strongly agree	Somewhat agree	Neither agree nor disagree	Somewhat disagree	Strongly disagree
I understand of the limitations of PGx.	○	○	○	○	○
I understand how drug-drug interactions may be altered in severity by genetic variability, and I know how to modify therapy.	○	○	○	○	○
I can describe cost of PGx testing and current reimbursement issues.	○	○	○	○	○
I know how to document PGx results and implications, and how to explain PGx results and implications of the information to patients.	○	○	○	○	○
I understand the ethical, legal, and social issues in the handling of PGx data.	○	○	○	○	○
I can communicate PGx to other healthcare providers.	○	○	○	○	○

Course QA

As a result of participating in the course "Applied PGx Education for the Healthcare Professional," . . .

	I expect to increase the number of PGx consultations I use in my practice.	I see the applicability of this information in my practice.	I will be able to use what I learned in this training in my practice in the next six months to a year.
Strongly agree			
Somewhat agree			
Neither agree nor disagree			
Somewhat disagree			
Strongly disagree			

What barriers in your clinical practice could you foresee hindering your ability to implement what you have learned?

How would you describe the pacing of the coursework?

- Too slow
- Just right
- Too fast

Which lecture(s) do you believe will be the most beneficial to your clinical practice?

Overall, did you find the homework to be beneficial to your learning?

- ○ Yes
- ○ [ ] No (please specify why)

Which homework was the most helpful?

[ ]

Which homework was the least helpful?

[ ]

Did you find the PGx ECHO session to be beneficial to your learning?

- ○ Definitely yes
- ○ Probably yes
- ○ Might or might not
- ○ Probably not
- ○ Definitely not

Would you attend additional PGx ECHO sessions?

- ○ Definitely yes
- ○ Probably yes
- ○ Might or might not
- ○ Probably not
- ○ Definitely not

Did you find the oral presentation you completed to be beneficial to your learning?

- Definitely yes
- Probably yes
- Might or might not
- Probably not
- Definitely not

Are there any topics that were not covered that you felt would be beneficial to your learning?

Is there anything else you would like to share about the course?

Powered by Qualtrics
