# Supplementary figures and images for: Pharmacist workforce training in pharmacogenomics with a focus on rural and underserved areas
Source: Front Genet. 2026 Mar 30;17:1794122. doi: 10.3389/fgene.2026.1794122 (PMC13070540; doi:10.3389/fgene.2026.1794122)

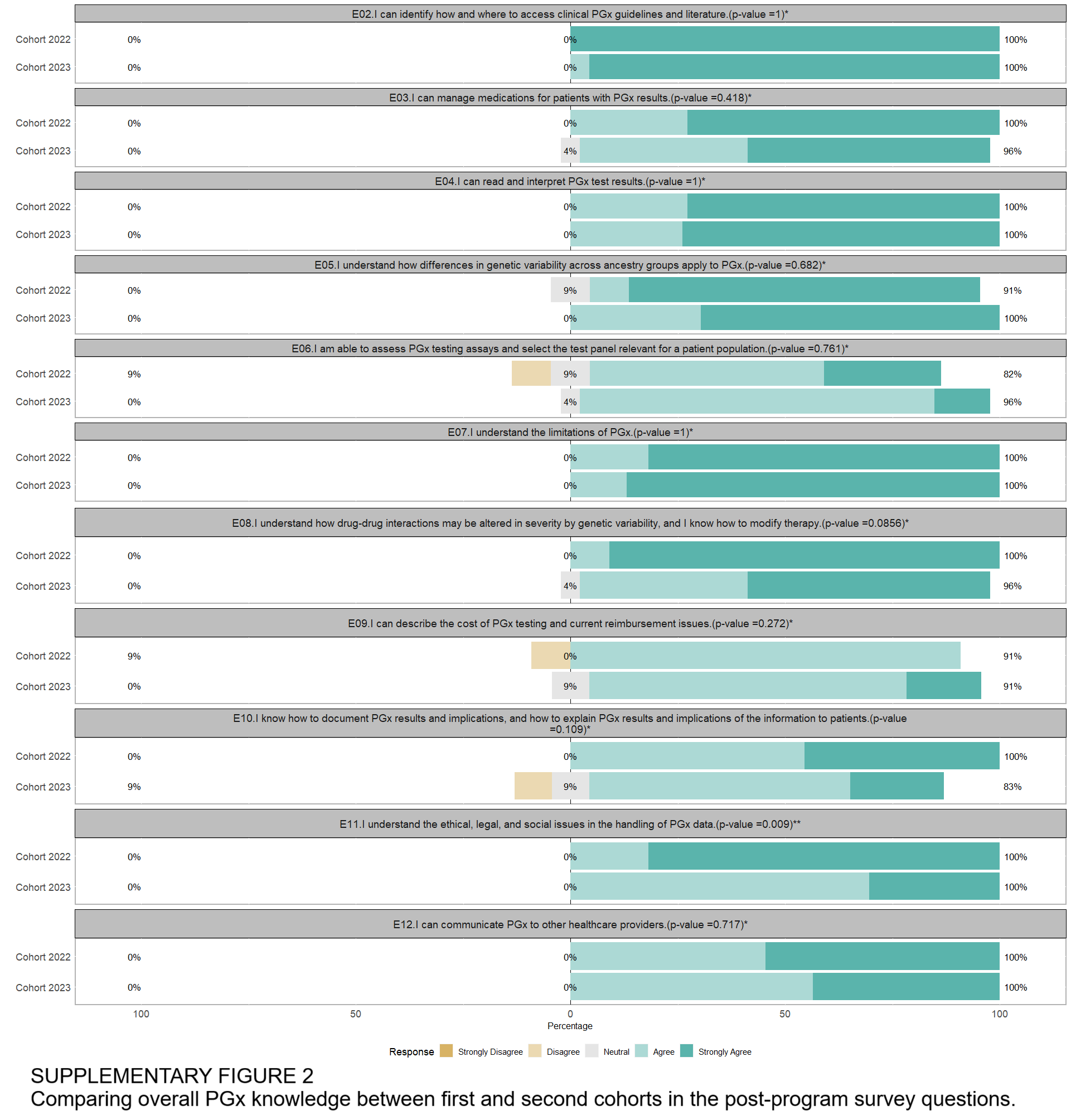

Supplement: Supplementary file 3 [file Image2.png]

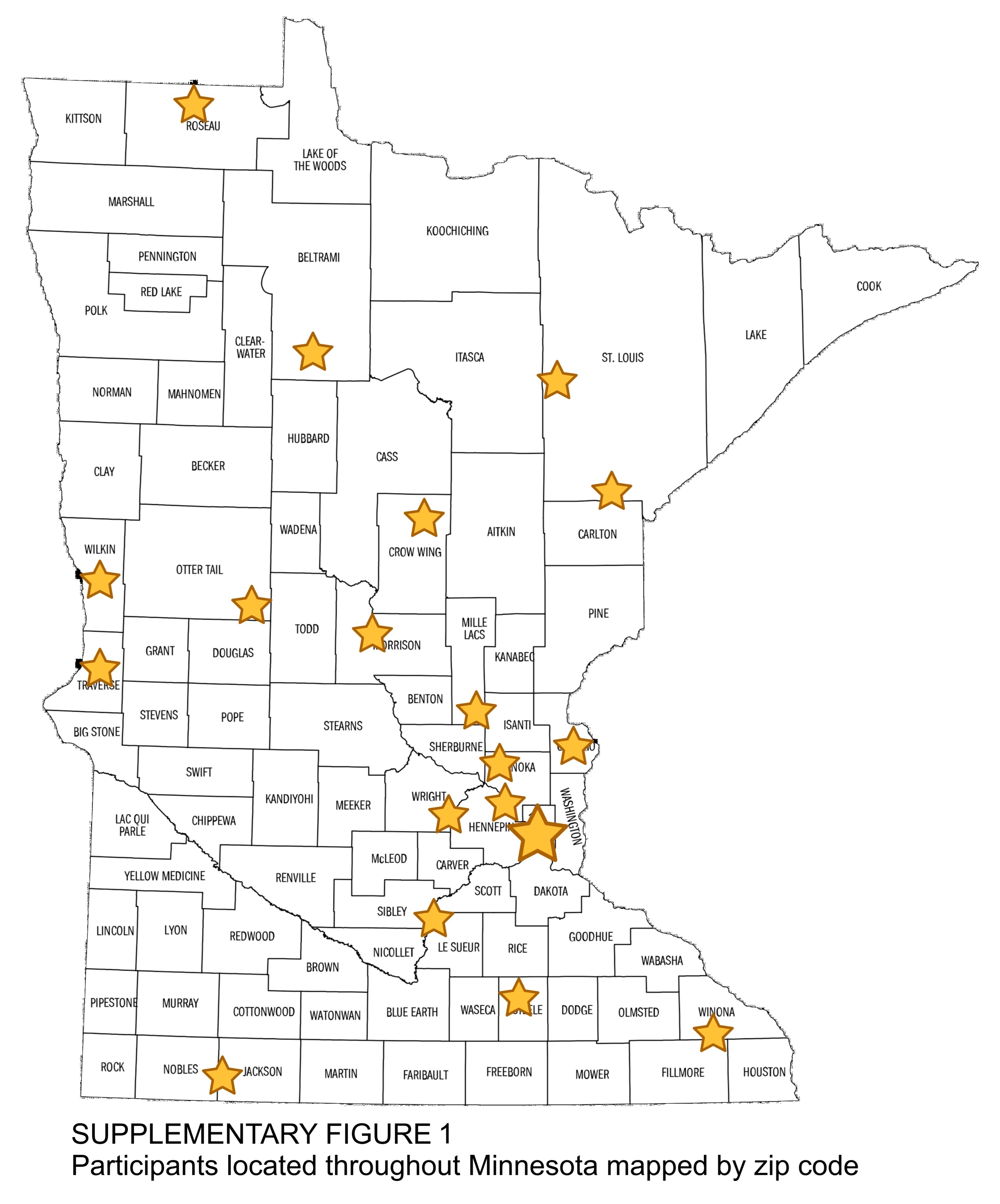

Supplement: Supplementary file 4 [file Image1.jpg]
